# Supplementary figures and images for: The epidemiology of antidepressant use in South Korea: Does short-term antidepressant use affect the relapse and recurrence of depressive episodes?
Source: PLoS One. 2019 Sep 25;14(9):e0222791. doi: 10.1371/journal.pone.0222791 (PMC6760791; doi:10.1371/journal.pone.0222791)

S1 Figure. Inclusion flow .

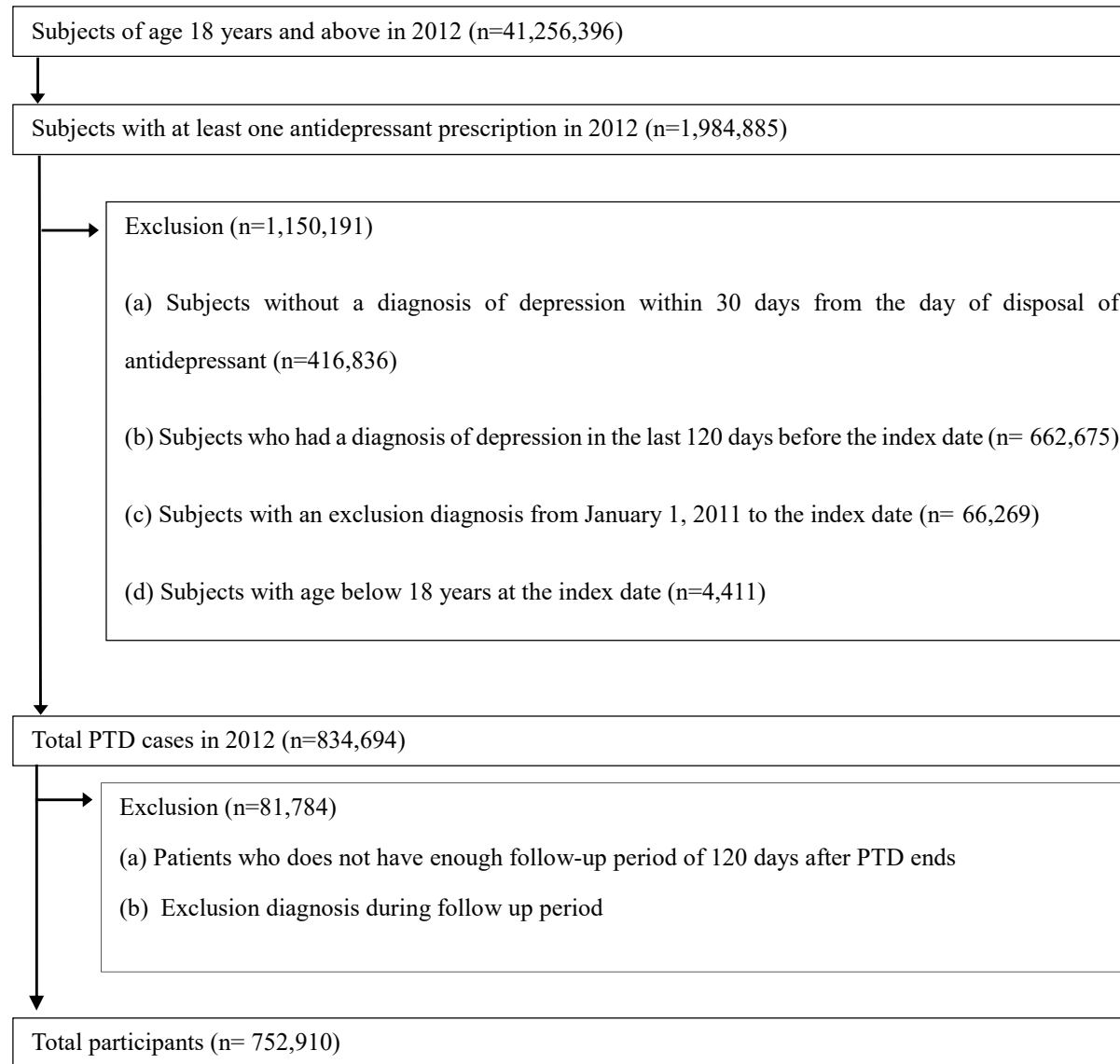

Supplement: S1 Fig — (PDF) [file pone.0222791.s001.pdf]
