# Supplementary material for: The epidemiology of antidepressant use in South Korea: Does short-term antidepressant use affect the relapse and recurrence of depressive episodes?
Source: PLoS One. 2019 Sep 25;14(9):e0222791. doi: 10.1371/journal.pone.0222791 (PMC6760791; doi:10.1371/journal.pone.0222791)
Supplement: S1 Table — (PDF) [file pone.0222791.s002.pdf]

S1 Table. The ICD-10 codes of psychiatric and non-psychiatric comorbidities

		ICD-10 code
Psychiatric comorbidities	Anxiety disorders	F40, F41, F43.1
	Substance related disorders	F10~ F19
	Obsessive-compulsive disorder	F42
	Personality disorders	F60~ F69
Non-psychiatric comorbidities	Cardiovascular diseases	I10~I15, I20~ I25, I47~I50,
	Diabetes mellitus	E10~E14
	Chronic obstructive pulmonary diseases	J40~J47
	Cancer	C00~C97, D00~D09, D10~D36, D37~D48
	Stroke	I60~I69
	Hypothyroidism	E00~ E03
