# Supplementary material for: The epidemiology of antidepressant use in South Korea: Does short-term antidepressant use affect the relapse and recurrence of depressive episodes?
Source: PLoS One. 2019 Sep 25;14(9):e0222791. doi: 10.1371/journal.pone.0222791 (PMC6760791; doi:10.1371/journal.pone.0222791)
Supplement: S2 Table — (PDF) [file pone.0222791.s003.pdf]

S2 Table. Index date prescription of antidepressants of inadequate and adequate antidepressant users in Korea

Characteristics	Total	Short-term user	Long-term user
**1 Drug N(%)**	424619 (92.70)	685704 (91.07)	261085 (88.55)
MAOIs	242 (0.03)	136 (0.03)	106 (0.04)
NaSSA	8,060 (1.07)	3,829 (0.83)	4,231 (1.43)
NDRI	7,092 (0.94)	3,699 (0.81)	3,393 (1.15)
SARI	37,354 (4.96)	21,422 (4.68)	15,932 (5.40)
SNRI	24,790 (3.29)	12,042 (2.63)	12,748 (4.32)
SSRI	174,877 (23.23)	88,441 (19.31)	86,436 (29.31)
TCA	432,291 (57.42)	294,300 (64.25)	137,991 (46.80)
Tetracyclic antidepressant	998 (0.13)	750 (0.16)	248 (0.08)
**2 Drugs (Same-Class Polypharmacy)**	28820 (6.29)	56495 (7.50)	27675 (9.39)
SSRI & SSRI	1,244 (0.17)	586 (0.13)	658 (0.22)
SSRI & SNRI	646 (0.09)	272 (0.06)	374 (0.13)
SSRI & TCA	15,896 (2.11)	8,503 (1.86)	7,393 (2.51)
SSRI & SARI	19,594 (2.60)	9,399 (2.05)	10,195 (3.46)
SSRI & NaSSA	1,009 (0.13)	402 (0.09)	607 (0.21)
SSRI & NDRI	777 (0.10)	354 (0.08)	423 (0.14)
TCA & TCA	6,181 (0.82)	3,861 (0.84)	2,320 (0.79)
SARI & TCA	3,779 (0.50)	2,007 (0.44)	1,772 (0.60)
SARI & SNRI	2,003 (0.27)	882 (0.19)	1,121 (0.38)
SNRI & TCA	2,754 (0.37)	1,332 (0.29)	1,422 (0.49)
Others	2,612 (0.35)	1,222 (0.27)	1,390 (0.47)
**2 Drugs (augmentation/ Multi-Class Polypharmacy)**	2865 (0.63)	6430 (0.85)	3565 (1.21)
MAOIs +APs	7 (0.00)	3 (0.00)	4 (0.00)
NaSSA +APs	421 (0.06)	174 (0.04)	247 (0.08)
NDRI+APs	167 (0.02)	73 (0.02)	94 (0.03)
SARI+APs	985 (0.13)	537 (0.12)	448 (0.15)
SNRI+APs	652 (0.09)	241 (0.05)	411 (0.14)
SSRI+APs	3,592 (0.48)	1,533 (0.33)	2,059 (0.70)
TCA+APs	606 (0.08)	304 (0.07)	302 (0.10)
Tetracyclic antidepressant+APs	0 (0)	0 (0)	0 (0)
**3 drugs**	1538 (0.34)	3734 (0.50)	2196 (0.74)
**More than 4 drug**	215 (0.05)	547 (0.07)	332 (0.11)
**Total**	752,910	458,057	294,853
